# Functional Burden and Quality of Life in Hip and Knee Osteoarthritis: A Cross-Sectional Study

**DOI:** 10.3390/medicina61071155

**Published:** 2025-06-26

**Authors:** Roxana Maria Sânziana Pavel, Anamaria Lavinia Purza, Delia Mirela Tit, Andrei-Flavius Radu, Diana Carina Iovanovici, Danche Vasileva, Bogdan Uivaraseanu, Gabriela Bungau, Carmen Delia Nistor-Cseppento

**Affiliations:** 1 Doctoral School of Biological and Biomedical Sciences, University of Oradea, 410087 Oradea, Romania; pavel.mariaroxanasanziana@student.uoradea.ro (R.M.S.P.); andreiflavius.radu@uoradea.ro (A.-F.R.); gbungau@uoradea.ro (G.B.); dcseppento@uoradea.ro (C.D.N.-C.); 2 Department of Pharmacy, Faculty of Medicine and Pharmacy, University of Oradea, 410028 Oradea, Romania; 3 Department of Psycho-Neurosciences and Recovery, Faculty of Medicine and Pharmacy, University of Oradea, 410073 Oradea, Romania; 4 Institute of Cardiovascular Diseases Timisoara, 300310 Timisoara, Romania; diana_iovanovici@yahoo.com; 5 Faculty of Medical Sciences, Goce Delcev University, 2000 Stip, North Macedonia; dance.vasileva@ugd.edu.mk; 6 Department of Surgical Disciplines, Faculty of Medicine and Pharmacy, University of Oradea, 410073 Oradea, Romania; buivaraseanu@uoradea.ro

**Keywords:** hip osteoarthritis, knee osteoarthritis, quality of life, activities of daily living, WOMAC index, WHOQOL-BREF

## Abstract

*Background and Objectives*: Osteoarthritis, the most common degenerative joint disease, causes pain, decreased mobility, and functional disability, having a significant impact on patients’ quality of life. This study aimed to evaluate the impact of hip osteoarthritis (HOA) and knee osteoarthritis (KOA) on physical functioning and quality of life, and to explore how these outcomes vary according to sex, disease stage, and common comorbidities. *Materials and Methods*: A cross-sectional study was conducted between 1 October and 30 December 2024, at the Medical Rehabilitation Department of Avram Iancu Clinical Hospital in Oradea, Romania. A total of 133 adult patients diagnosed with HOA or KOA, based on clinical and radiographic criteria, were included. Functional status was assessed using the Western Ontario and McMaster Universities Osteoarthritis Index (WOMAC), while quality of life was evaluated using the World Health Organization quality of life questionnaire-BREF version (WHOQOL-BREF). The main outcomes were the total scores of these instruments, analyzed in relation to demographic and clinical variables. *Results*: Based on the clinical staging criteria applied in the study, 23 patients (17.3%) were classified as being in the early stage, 98 (73.7%) in the progressive stage, and 12 (9.0%) in the advanced or end stage of the disease. The mean WOMAC total score was 52.0 ± 7.9 (scale: 0–96), indicating moderate to severe functional impairment. The mean WHOQOL-BREF score was 67.9 ± 13.1 (scale: 0–100), reflecting a moderately reduced quality of life. A moderate, statistically significant inverse correlation was observed between WOMAC and WHOQOL-BREF scores (Spearman’s rho = −0.565, *p* < 0.001). Patients with knee osteoarthritis reported significantly lower quality of life compared to those without this condition (66.48 ± 12.73 vs. 71.76 ± 13.31, *p* = 0.006). No statistically significant differences were found in functional, or quality-of-life scores based on sex. *Conclusions*: Knee osteoarthritis, particularly when combined with hip involvement, is associated with a substantial decline in quality of life and functional capacity. The severity and location of joint involvement appear to be the primary determinants of disability in this patient population, while systemic comorbidities have a less pronounced influence in the rehabilitation setting.

## 1. Introduction

Osteoarthritis (OA) is the most common degenerative joint disease, mainly affecting weight-bearing joints such as the hips and knees. It is a major cause of pain, reduced mobility and functional disability among the adult and elderly population, with a significant impact on the quality of life (QoL) of patients [1,2]. Hip osteoarthritis (HOA) and knee osteoarthritis (KOA) are the most common forms of OA and are frequently associated with decreased self-care capacity, social isolation, and affective disorders [3], significantly reducing QoL and representing the leading cause of walking disability, particularly in the knee and hip [4].

OA impacts around 528 million individuals globally. The prevalence is 23% among the global population aged 40 and above. The knee is the most frequently impacted joint, involved in roughly 60% to 85% of all OA occurrences [5]. The growing prevalence of OA is largely attributed to an aging population and the increasing burden of risk factors such as obesity, physical inactivity, and metabolic comorbidities. Although OA is often viewed as a slowly progressing disease, its impact on functionality and psychosocial well-being can be profound and rapidly progressive [6].

HOA and KOA are responsible for a significant burden in terms of years lived with disability, with a considerable economic impact of up to 2.5% of the gross domestic product in high-income countries, primarily due to the intensive use of healthcare services, including surgical interventions like arthroplasty. Furthermore, the disease also affects the social and economic aspects of patients’ lives, leading to absenteeism, early retirement, and decreased productivity. Functional performance limitations have negative effects on mental health, QoL, and participation in social, recreational, and occupational activities. The long and fluctuating course of the disease, characterized by a complex and variable process with symptoms and progression that differ from patient to patient, makes it difficult to predict the disease’s trajectory and its long-term impact on physical function and QoL [7].

OA arises from a complex interplay of contributing factors and involves progressive structural deterioration, notably affecting articular cartilage, inducing synovial membrane inflammation, and prompting subchondral bone remodeling marked by osteophyte development [8]. A growing body of research emphasizes the dynamic interaction between biomechanical stress affecting the osteochondral interface and persistent, low-grade inflammation within the synovial tissue, known as synovitis, as key contributors to the pathophysiological mechanisms underpinning OA [9]. Clinical manifestations such as joint pain, morning stiffness, reduced range of motion, muscle weakness, fatigue, and gait abnormalities tend to progressively intensify, ultimately contributing to significant functional impairment and disability [10].

Despite advancements in understanding its pathogenesis, no current therapeutic option has demonstrated efficacy in halting or reversing disease progression. Management strategies remain confined to symptom control through both medical and non-medical interventions, with surgical procedures reserved for advanced or refractory cases [8].

In recent years, the QoL has become an essential indicator in the assessment of the health status of patients with chronic diseases. Standardized tools, such as the WHO Quality of Life-BREF (WHOQOL-BREF, developed by the World Health Organization), allow a multidimensional assessment of the subjective state of patients, analyzing the physical, psychological, social dimensions and the relationship with the living environment [11]. Additionally, the Western Ontario and McMaster Universities Osteoarthritis Index (WOMAC) is used extensively in research and clinical practice to assess pain, stiffness, and physical function in patients with hip or knee OA, due to its proven psychometric properties [12,13].

Available studies have highlighted the existence of frequent discrepancies between the severity of reported symptoms and the structural changes evidenced by imaging, which makes subjective assessment tools essential in understanding the real impact of the disease on patients [14]. However, in Romania, integrative assessments of the QoL in the context of OA, especially within medical rehabilitation services, remain limited.

**Figure 1:**
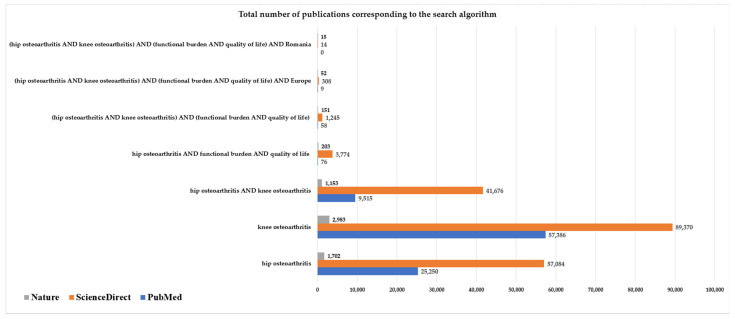
Database exploration of HOA and KOA with respect to QoL and functional impact using Boolean operators.

In the initial stage, a structured literature search, non-limited to original research articles (Figure 1), was conducted using key terms such as HOA and KOA, and used singularly or in combination with terms like functional burden and quality of life, employing basic Boolean operators (AND, OR, NOT). While the overall number of publications on HOA or KOA individually is substantial, studies specifically addressing the combined impact of both conditions on functional status and quality of life are significantly fewer. Our search across major academic databases with strong medical coverage (i.e., PubMed, ScienceDirect, and Nature) revealed notable disparities in both the geographic distribution and the volume of publications within this specific field. For example, the search algorithm including “Romania” along with “hip osteoarthritis AND knee osteoarthritis AND functional burden AND quality of life” returned either no results or publications unrelated to the objectives of our study. None of the results displayed by the search containing the search algorithm ‘(hip osteoarthritis AND knee osteoarthritis) AND (functional burden AND quality of life) AND Romania’ aligned with our research focus in terms of design, population, or outcome measures.

Similarly, searches incorporating broader regional terms such as “Europe” produced a modest increase in the number of results, predominantly from Western European countries; however, these studies do not fully align with the specific methodological framework or outcome focus of the present research, thereby reinforcing the need for continued investigation into the combined impact of KOA and HOA on functional burden and QoL. Therefore, studies that simultaneously explore both HOA and KOA together with measures of functional burden and quality of life, the exact focus of our investigation, are extremely rare. This highlights a clear gap in the literature and underscores the relevance and originality of our study.

There is growing evidence that population-level differences across Europe influence health outcomes and care delivery, with contributing factors spanning from genetic variability to socioeconomic conditions [15,16]. At the genetic level, multiple single nucleotide polymorphisms exhibit significant variation among European populations, with the extent of divergence often correlating with geographic distance. Some of these disparities appear to be driven by natural selection, suggesting an evolutionary advantage for specific alleles in certain regions [15,17]. Beyond genetics, substantial differences are also observed in health care access and the organization of medical services. Rehabilitation services, for example, vary widely in structure and specialization across European countries, with many systems favoring outpatient models and offering limited indication-specific facilities [18]. Socioeconomic status adds another layer of complexity. Individual-level analyses consistently demonstrate the existence of health inequalities associated with income across the continent. At a macro level, countries with greater income disparities tend to report higher levels of health inequality [16].

Existing research highlights that European populations differ across multiple layers, including genetic, socioeconomic, and healthcare-related dimensions. These differences influence not only disease perception and treatment practices but also broader health outcomes. Given the complex and layered nature of these disparities, it is essential that studies continue to investigate these variations systematically. Research efforts should be tailored to specific population groups, recognizing that regional, social, functional and biological factors may shape health behaviors and responses to care in distinct ways.

OA continues to impose a substantial functional and societal burden, yet most studies have narrowly focused on structural or pharmacologic outcomes, with limited emphasis on how OA impacts daily functioning and quality of life, particularly in rehabilitation contexts where conservative treatment dominates. Moreover, nearly all such research has been concentrated in Western settings, overlooking populations in Eastern Europe whose experiences are shaped by distinct healthcare infrastructures, socioeconomic challenges, and cultural perceptions of chronic illness. Romania exemplifies this gap: although OA prevalence is high, there is a near-absence of studies that assess how patients functionally experience simultaneously KOA and HOA, and how these experiences correlate with QoL using validated, multidimensional tools. In Romanian populations, OA is frequently perceived as a natural and inevitable aspect of aging, a belief that may influence treatment-seeking behavior. Traditional remedies and natural treatments are commonly prioritized over conventional medical interventions. Furthermore, treatment choices are often shaped by social influences, including the attitudes and experiences of family members and the broader community, emphasizing the role of interpersonal and cultural factors in pain management and care pathways.

While previous studies have employed standard patient-reported outcome measures and multivariate analyses to assess QoL in arthritis populations, few have explored how these outcomes manifest in under-resourced, post-communist healthcare systems. Therefore, the present study aims to address through a multidimensional assessment the above-mentioned knowledge gaps in research by investigating the combined functional and QoL impact of KOA and HOA in a Romanian rehabilitation setting, a population and context rarely represented in current scientific literature. These findings may help enrich the broader European understanding of the functional and QoL impact of KOA and HOA within diverse healthcare contexts and models.

## 2. Materials and Methods

### 2.1. Study Design

This cross-sectional study was conducted between 1 October and 30 December 2024, in the Medical Rehabilitation Department of Avram Iancu Clinical Hospital in Oradea, Romania. The primary aim was to assess the QoL in patients diagnosed with HOA and KOA. Diagnosis was established by a certified specialist in physical and rehabilitation medicine, based on clinical examination and standard radiographic imaging, in accordance with the diagnostic criteria of the American College of Rheumatology (ACR). A total of 138 patients were initially evaluated, out of which 133 met the inclusion criteria and agreed to participate in the study.

The severity of OA was determined through clinical examination and pelvic radiographs, based on joint morphology and degenerative changes. Patients were classified into four stages of disease progression, as described in previously published guidelines [15]. Stage I included early signs such as minor joint space narrowing, often without clear clinical symptoms. Stage II involved visible narrowing of the joint space and mild hardening of the subchondral bone. In stage III, joint deterioration became more evident, with moderate narrowing, the appearance of small bone spurs, and early joint surface irregularities. Stage IV was characterized by advanced structural damage, including joint space collapse, large osteophytes, bone cysts, and significant deformities affecting joint function. In this study, we considered stages I and II as early stage, as they represent initial phases with minimal or absent signs of joint damage. Stage III, referred to as progressive, indicates clearer but still non-destructive deterioration, while stage IV, classified as advanced, represents the final stage, with severe damage and major structural changes affecting joint function. We chose this classification to better analyze the impact of each stage on patients’ functionality and QoL, in line with our objective of identifying the factors that contribute most to deterioration and the decline in perceived health status. Given that most participants presented with bilateral joint involvement, particularly in moderate to advanced stages of OA, we opted to classify each patient based on the most severely affected joint. This methodological decision is supported by the clinical observation that the joint with the greatest structural damage most often dictates functional limitations and overall disability perception. This approach also aligns with similar methodologies in OA studies focusing on patient-centered outcomes, where the dominant symptomatic joint is considered the primary determinant of QoL and functional impact [19,20].

Functional assessment using the WOMAC Index and health-related QoL evaluation through the WHOQOL-BREF questionnaire were performed at the time of admission by trained physiotherapists. All evaluations were supervised by the attending rehabilitation physician to ensure consistency and accuracy.

### 2.2. Inclusion/Exclusion Criteria

**Figure 2:**
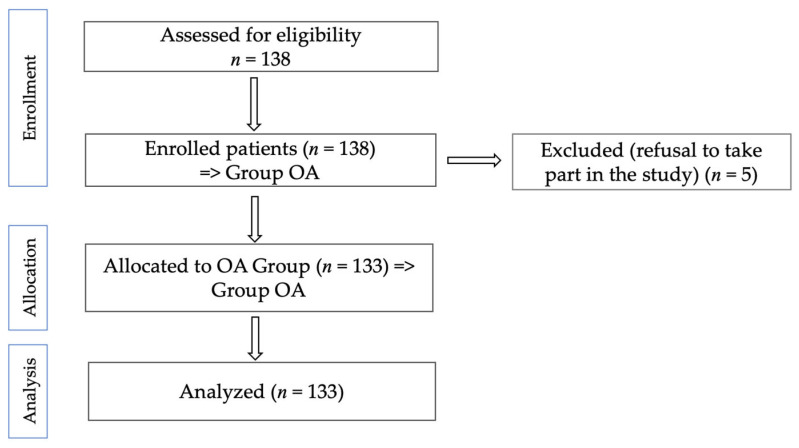
CONSORT flow diagram of the present study.

All patients admitted to the rehabilitation department during the study period with a confirmed diagnosis of degenerative hip or knee pathology were considered eligible, regardless of disease stage. Individuals were excluded if they declined participation or presented with neurological conditions, such as balance disorders, spasticity, or post-stroke rigidity, that could hinder proper functional assessment. After applying these criteria, 133 patients were included in the final sample. The recruitment process is outlined in the CONSORT diagram (Figure 2).

### 2.3. Sample Size

The sample size calculation for this study was based on the total number of patients who visited the outpatient rehabilitation department during the defined study period. To ensure the representativeness and validity of the sample, we applied a standard formula commonly used in prevalence studies where the outcome is binary (e.g., presence or absence of disease) [21].

n = t^2^ pq/(x^2^ + t^2^ pq/N)

where p refers to the estimated probability of the phenomenon (here, functional limitation due to OA), q = 1 − p, t is the Z-score corresponding to the desired confidence level (1.96 for 95%), x is the accepted margin of error, and N represents the total population size. For our study, we adopted a conservative approach by setting up at 0.5 to maximize sample variability, standard practice when there is no prior estimate of prevalence. A margin of error of 10% (x = 0.1) and a confidence level of 95% (t = 1.96) were chosen, and the total population (N) was approximately 1256 patients. Using this method, the minimum required sample size was calculated to be 96 participants.

### 2.4. Study Tools

The clinical severity was evaluated using the WOMAC Index, a validated and widely used instrument available in the Romanian language. Specifically designed for patients with HOA and KOA, the WOMAC questionnaire consists of 24 items grouped into three subscales: pain (5 items), stiffness (2 items), and physical function (17 items). Each item is scored on a 5-point Likert scale ranging from 0 (no symptoms) to 4 (extreme symptoms), with higher scores reflecting greater severity. The pain subscale evaluates discomfort during common activities such as walking, stair climbing, standing, sitting, and resting. The stiffness subscale addresses joint stiffness upon waking and throughout the day. The physical function subscale captures limitations in daily living activities, such as dressing, bending, or performing household tasks. The maximum possible total score is 96, calculated as the sum of all subscale scores, and represents the overall burden of disease in terms of pain, stiffness, and disability [22].

To assess health-related quality of life (HRQoL), we used the WHOQOL-BREF, the abbreviated version of the original WHOQOL instrument developed by the World Health Organization. This tool includes 26 items, two of which explore general perceptions of QoL and health (Q1 and Q2). The remaining 24 items are grouped into four domains: physical health, psychological well-being, social relationships, and environment. Responses are rated on a 5-point Likert scale, where participants assess their experience from “very poor” (1) to “very good” (5). This applies to both general perception items and domain-specific questions. The scores are then transformed to a 0–100 scale, with higher values indicating better perceived QoL. This structure allows for both domain-specific and overall assessment of the patient’s subjective health status [23]. In this study, only the WHOQOL-BREF total score was used, as it provides a global, integrative measure of the patient’s perception of their well-being and allows a more direct and unified interpretation of the relationship with the clinical factors investigated, such as symptom severity or the presence of comorbidities.

Both instruments applied in this study have been previously translated and validated for use in Romanian populations. Although a formal validation study for the Romanian version of WOMAC is not currently available in the published literature, its use is supported by official listings from the developers, which confirm linguistic validation for Romanian-speaking populations [24]. This version has been widely applied in both clinical and epidemiological studies involving musculoskeletal disorders in Romania. Similarly, the WHOQOL-BREF has been officially adapted by the World Health Organization for Romanian speakers, with acceptable psychometric properties in diverse health settings. Their use in this study ensures cultural and linguistic appropriateness for the target population [25].

### 2.5. Ethical Approval

The study was approved by the Ethics Commission “Avram Iancu” Clinical Hospital from Oradea, Romania (approval no. 4842/03.12.2020) and complies with the Declaration of Helsinki of the World Medical Association. Written informed consent was obtained from all participants prior to data collection.

### 2.6. Statistical Analysis

Data were analyzed using JASP software (version 0.19.3.0). Continuous variables were summarized using medians and interquartile ranges where non-normality was identified based on the Shapiro–Wilk test, while categorical data were reported as absolute and relative frequencies. The Levene test was used to assess the homogeneity of variances. Given that most continuous variables did not meet the assumptions of normal distribution, non-parametric tests were applied throughout the analysis.

Comparisons between groups (e.g., by sex or disease stage) were performed using the Mann–Whitney U test and Kruskal–Wallis test, as appropriate. When statistically significant differences were identified in the Kruskal–Wallis test, post hoc pairwise comparisons were performed using Dunn’s test, with the Holm correction applied to adjust for multiple testing. Associations between clinical variables and outcome measures (WOMAC and WHOQOL-BREF total score) were explored through Spearman’s rank correlation and multiple linear regression analysis. A significance threshold of *p* < 0.05 was applied in all tests.

## 3. Results

### 3.1. General Characteristics of the Cohort

The study included 133 patients diagnosed with primary OA of large joints. The mean age of the patients was 63.4 ± 9.8 years, ranging between 39 and 82 years. The gender distribution was relatively balanced (63 women—47.4% and 70 men—52.6%). Most patients were from urban areas (71.4%), with the remaining ~28.6% living in rural areas. In terms of weight status, patients had a mean body mass index (BMI) of 32.2 ± 6.9 kg/m^2^, indicating moderate obesity at the group level. Over 80% of subjects (86.5%) were overweight or obese (BMI ≥ 25 kg/m^2^), including 67.7% with defined obesity (BMI ≥ 30). The main associated comorbidities were hypertension (present in 79.7% of patients), peripheral varicose disease (48.9%), type 2 DM (30.8%) and chronic kidney disease (CKD) (19.5%).

Regarding the distribution of arthritic disease, 64 patients (48.1%) had concurrent bilateral or contralateral hip and knee impairment, while 34 (25.6%) had isolated HOA, and 32 (24.1%) had isolated KOA. Clinical staging of OA (according to the criteria used in the study) indicated that 23 patients (17.3%) were in the early stage, 98 (73.7%) in the progressive stage of the disease, and 12 patients (9.0%) in the advanced/end stage. Table 1 summarizes the basic demographic and clinical characteristics of the study group.

### 3.2. Assessment of Functional Status (WOMAC) and Quality of Life (WHOQOL-BREF Total)

The WOMAC total score had a mean of 52.0 ± 7.9 (on a scale of 0–96, where higher values indicate a more severe condition) and a median of 52 points, with a range between 36 (minimum, milder symptoms) and 67 (maximum observed, more severe symptoms). These values indicate a moderate to severe level of functional impairment and pain among patients, without reaching, however, the maximum theoretical values of the scale (no patient reported scores above 70 or close to 96).

By examining the WOMAC sub-scores (Table 2), it is noted that the reported joint pain has an average of 7.8 ± 2.0 (out of 20 possible points), which corresponds to a moderate pain level on average. The minimum pain sub score value was 4/20, indicating that even the least affected patients experience some joint discomfort. Joint stiffness (morning stiffness) averaged 5.8 ± 1.4 on the WOMAC subscale (0–8), with a median of 6, indicating the presence of a moderate stiffness sensation. Physical function (ability to perform daily activities) was the most affected component: the WOMAC subscore for function had a mean of 38.4 ± 5.6 (out of 68 possible points), with a median of 38 (range 30–48). This mean value indicates significant functional limitations among the patients. In particular, no patient reported complete independence (minimum observed functional score 30 out of 68), suggesting that all subjects had difficulty with certain physical activities (such as walking, climbing stairs, squatting, etc.).

QoL assessed by the WHOQOL-BREF questionnaire was generally moderately impaired. The total WHOQOL-BREF score had a mean value of 67.9 ± 13.1 (on a scale of 0–100, where higher values indicate better QoL). Individual total score values varied over a wide range (minimum 19, maximum 89), with a median of 72 (Table 2).

**Figure 3:**
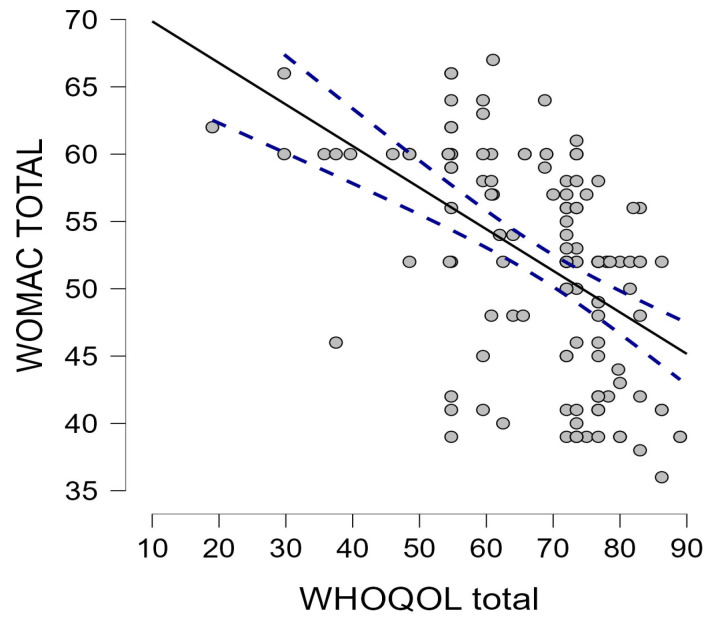
Inverse correlation between WOMAC total score and WHOQOL-BREF total score.

To explore the direct relationship between QoL and symptom severity, the correlation between the quality of life (WHOQOL-BREF) total score and the OA severity (WOMAC) total score was examined. A moderate and statistically significant inverse correlation was found between the two variables (Spearman’s rho = −0.565, *p* < 0.001) (Figure 3). This indicates that, in general, patients who report greater pain and functional limitations (higher WOMAC score) tend to report a lower quality of life (lower WHOQOL score) and vice versa, suggesting a symptomatic burden of OA with a direct, quantifiable impact on the well-being of patients in their daily lives.

**Figure 4:**
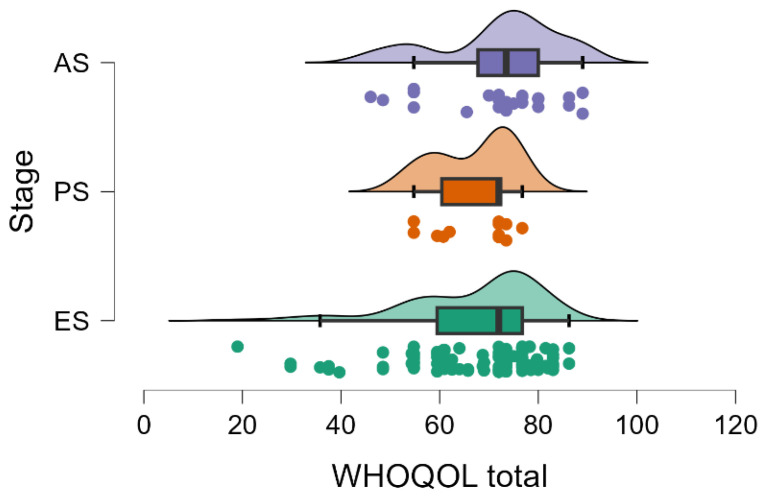
Distribution of total WOMAC scores according to osteoarthritis stage. ES—early stage (I and II); PS—progressive stage (III); AS—advanced/end-stage (IV).

In addition, it was investigated whether QoL is perceived differently between clinical severity subgroups. ANOVA analysis indicated significant differences between WOMAC total scores according to disease stage (F(2,130) = 8.46, *p* < 0.001), where F represents the test statistic, and the numbers in parentheses correspond to the degrees of freedom: 2 for between-group comparisons (three stages of disease minus one) and 130 for within-group variation (total sample minus the number of groups). These results suggest that symptom severity and functional limitations increase with OA progression. Patients in the early stage had the lowest scores (mean = 46.35), reflecting a lower degree of dysfunction, while scores were higher in the advanced (mean = 53.43) and final (mean = 50.83) stages (Figure 4). The non-parametric Kruskal–Wallis test confirmed these differences (H(2) = 13.17, *p* = 0.001), where H is the test statistic and the number in parentheses indicates the degrees of freedom (number of groups minus one). Post-hoc Dunn analysis showed that the significant difference occurs between the early and advanced stages (*p* < 0.001). There were no significant differences between the advanced and final stages, which could reflect either an adaptation effect to symptoms or already initiated therapeutic responses.

**Figure 5:**
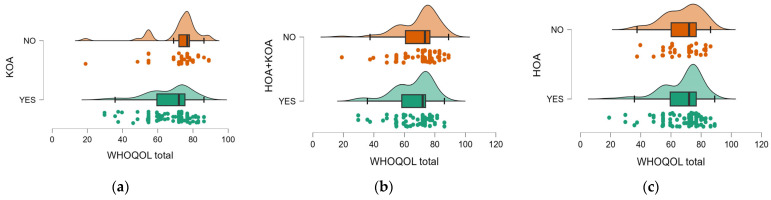
Distribution of total WOMAC scores according to OA location (**a**) KOA, (**b**) HOA + KOA, (**c**) HOA.

Analysis of the WHOQOL-BREF total score according to OA location showed that patients with KOA reported a significantly lower QoL compared to those without this condition (66.48 ± 12.73 vs. 71.76 ± 13.31, *p* = 0.006) (Figure 5a). Also, patients with concurrent hip and knee impairment had significantly lower WHOQOL scores than those with single impairment (65.93 ± 12.91 vs. 69.82 ± 13.0, *p* = 0.043) (Figure 5b). In contrast, patients with isolated HOA did not show a significant difference from others in terms of self-reported QoL (67.99 ± 13.35 vs. 67.83 ± 12.42, *p* = 0.754) (Figure 5c).

### 3.3. Assessing Differences by Sex

No significant differences were identified between the sexes in terms of mean age (62.6 ± 10.6 years in men vs. 64.3 ± 8.9 years in women, *p* = 0.326) or body mass index (32.5 ± 5.8 vs. 31.9 ± 7.8, *p* = 0.327). Analysis of chronic comorbidities revealed similar rates between the two sexes. Type 2 DM was present in 37.1% of men and 44.4% of women (*p* = 0.392), and hypertension in over three quarters of cases in both groups. Although no significant differences were recorded, there is a trend for CKD and venous insufficiency to be slightly more common in men, while diabetes appears to be more prevalent in women (Table 3).

The prevalence of hip and KOA was also similar between the sexes. HOA was slightly more common in women (79.4%) than in men (68.6%), and KOA was more common in men (77.1% vs. 66.7%), but the differences were not statistically significant (*p* > 0.05). The concurrent impairment of both large joints was almost identically distributed: 48.6% in men and 47.6% in women (*p* = 0.983).

Regarding functional status, women had a mean WOMAC total score of 52.9 ± 7.6, compared with 51.1 ± 8.2 in men (*p* = 0.20). Similarly, no individual component of the scores differed significantly by sex: the WOMAC subdomains (pain, stiffness, function) had similar mean values in women versus men (all gender comparisons had *p* > 0.05). The quality of life (WHOQOL-BREF) total score was slightly lower in women (66.4 ± 13.2) than in men (69.3 ± 13.0), but this difference did not reach statistical significance (*p* = 0.20). Therefore, in the studied cohort, the impact of OA on functional status and QoL seems to be similar in female and male patients (Table 4).

**Figure 6:**
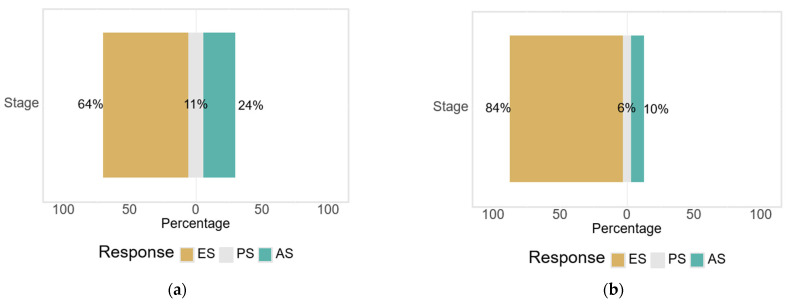
Distribution of degenerative hip and knee pathology according to disease stage and sex: (**a**) men; and (**b**) women.

The only significant difference between sexes was observed in the distribution of clinical stages of OA (Figure 6). A significantly higher percentage of women were in the progressive stage (84.1% vs. 64.3% in men), while men were more frequently represented in the early stages and advanced stages (*p* = 0.032), suggesting a possible variation in disease progression or presentation behavior.

### 3.4. Predictors of WOMAC Score

To identify the factors influencing the clinical severity of OA, we performed a multiple linear regression analysis, having as dependent variable the WOMAC total score and as main independent variables: age, sex, body mass index (BMI), stage of the disease, concurrent joint impairment (KOA + HOA), as well as the presence of common comorbidities (type 2 diabetes mellites (DM), peripheral varicose disease, hypertension and CKD).

The overall regression model was statistically significant (F (10,122) = 3.611, *p* < 0.001) and explained approximately 16.5% of the variability in the WOMAC score (adjusted R^2^ = 0.165), suggesting a modest, but clinically relevant, influence of the analyzed factors on the perceived severity of symptoms.

Among the variables included, the only predictor with a significant and consistent effect was the presence of concurrent hip and knee joint impairment. Patients without this concurrent impairment had significantly lower WOMAC scores by almost 5 points (β = −4.99, *p* < 0.001), highlighting the cumulative impact of polyarticular involvement on physical dysfunction and pain. Also, patients in the early stage of the disease presented significantly lower WOMAC scores compared to those in the progressive stage, with a mean difference of 6.3 points (*p* = 0.001). This confirms a clear relationship between disease progression and worsening of symptoms.

In contrast, age, sex, BMI and the other comorbidities that were analyzed (DM, HBP, CKD, varicose veins) did not demonstrate a significant predictive effect on the WOMAC score in the final model. Although at a descriptive level older patients or those with comorbidities appeared to have higher WOMAC scores, these relationships did not remain significant after adjustment for other factors. This result suggests that, in the studied clinical context, disease location and stage are the main determinants of perceived dysfunction, while systemic factors may have a more attenuated or indirect influence. The detailed results of the multiple regression analysis are presented in Table 5.

## 4. Discussion

This study examined the extent to which HOA and KOA compromises functional status and perceived QoL in patients undergoing rehabilitation care. Based on prior evidence suggesting a multidimensional burden of OA, it was anticipated that these outcomes would be interrelated and potentially influenced by clinical and demographic variables such as joint location, disease stage, comorbidities, and sex. The findings confirmed a moderate to severe level of pain and physical limitation, as indicated by WOMAC scores, and a moderate reduction in perceived QoL, as measured by WHOQOL-BREF.

A significant inverse correlation between functional impairment and QoL supports the notion that symptom severity directly affects patients’ subjective well-being. Among the examined variables, disease location and severity showed the strongest associations with outcome scores, while the influence of sex and comorbidities appeared to be less pronounced within this cohort.

These findings align with previous research indicating that OA, particularly when affecting weight-bearing joints, substantially impairs both physical function and QoL [26,27,28,29], even among patients actively engaged in rehabilitation. The persistence of symptoms despite ongoing care highlights the chronic and multidimensional burden of the disease.

The average age of patients included in this study was 63.4 years, consistent with data from the literature, which indicates a high prevalence of OA in people over 55 years of age, with over 70% of older adults being affected by this disease at some point, according to the World Health Organization [30]. Although numerous epidemiological studies have shown that OA is more common in women, especially after menopause, when hormonal changes can influence cartilage integrity [1,31]. In our study group, the sex distribution was relatively balanced (women: 47.4%; men: 52.6%). This aspect could reflect the specifics of the population that addresses medical rehabilitation services, where the level of dysfunction and pain determine a comparable need for treatment in both groups. Similar data were also reported in the European Project on Osteoarthritis study [32], which emphasized that gender differences in access to rehabilitation are less pronounced in clinical settings compared to population-based studies, likely due to variations in healthcare-seeking behavior and service availability.

Approximately half of the patients in our cohort had concurrent hip and KOA, a proportion somewhat lower than that reported by Fekete et al. (2021), who found that nearly 75% of patients exhibited concurrent involvement of both joints [33]. This discrepancy may be attributed to differences in recruitment settings. In our study, participants were enrolled from a physical medicine and rehabilitation department, where individuals with single-joint involvement, often in earlier or less complex stages, are more likely to seek care. In contrast, studies recruiting from orthopedic, or rheumatology services often reflect a higher proportion of advanced, multi-joint disease, given the greater clinical severity and surgical referral patterns in those contexts.

The WOMAC index is widely recognized for its psychometric robustness and is frequently used to evaluate clinical status in patients with HOA and KOA. Its structure enables reliable and sensitive measurement of both pain and functional limitation, offering clinicians a validated tool for tracking symptom severity and response to treatment [34,35]. The pain and physical function subscales, in particular, allow for high-precision assessments that are responsive to therapeutic interventions and reflective of real-world limitations.

In this cohort, the degree of functional impairment revealed by the WOMAC scores was significant, with an average of approximately 52/96 placing the patients included in this study in a moderate disability area. Practically, patients suffering from hip and knee OA experience significant difficulties in performing daily activities (moving, climbing stairs, getting up from a chair, etc.), a fact highlighted by the WOMAC physical function subscore which reached approximately 56% of the maximum possible deficits. WHOQOL-BREF scores around 68/100 indicate a moderately reduced QoL, confirming that OA generates dysfunctions and limitations with multidimensional benchmarks. These findings are in agreement with the literature, which shows that OA causes a marked reduction in physical capabilities and perceived health status in the elderly [36]. Although many patients in our study presented with bilateral joint involvement, we focused the analysis on the most severely affected joint. While this might slightly accentuate perceived symptom severity, it offers a clinically relevant estimation of functional burden, as patients commonly report limitations based on the joint causing the most discomfort [19,20,37].

Recent research further supports the link between symptom intensity and QoL, with increased pain and stiffness in KOA being associated with reduced health perception across all WHOQOL-BREF domains [37]. Our results reinforce these observations, highlighting a significant inverse correlation between WOMAC score and QoL. Patients who suffer more from OA also rate their QoL as poorer. This fact emphasizes the importance of optimal symptomatic treatment (analgesics, anti-inflammatories, physiotherapy, etc.) [38] and rehabilitation interventions in the management of OA, as pain relief and improved physical function could have a direct positive effect on the overall well-being of patients [37]. The therapeutic approach in OA must be multidisciplinary, targeting not only the joint itself, but also the functional and psychosocial consequences of the disease, to prevent the decline in the QoL [39].

In this study, we observed no significant gender differences in WOMAC or QoL outcomes, despite women presenting with more advanced radiographic disease. This contrasts with some earlier studies in which female OA patients reported worse pain and QoL than men [26,40]. The discrepancy may reflect our study setting, both sexes had similarly high symptom burdens, perhaps due to the predominance of PS disease in each group. It is possible that cultural or healthcare-seeking factors led to later presentation in women, as a markedly higher proportion of our female patients had PS OA. In other populations, women experience more severe OA-related disability [41], but our data suggest that when disease severity is comparable, sex differences in symptom reporting may be attenuated. This underscores the need for early detection and equitable access to care, which may influence both clinical presentation and treatment outcomes.

Regarding the determinants of OA severity, the multivariate analysis applied to the group included in this study suggested that concurrent hip and knee impairment is a major factor in worsening the disability. Patients with polyarticular disease had WOMAC scores approximately 5 points (10%) higher than those with OA localized at a single joint. This result is determined by the fact that the involvement of two weight-bearing joints creates a biomechanical vicious circle. Pain in one hip or knee overloads the contralateral joint, and the limitation of mobility in one limb puts additional stress on the other, amplifying the overall dysfunction. To our knowledge, only a few studies have precisely quantified the difference in impact between mono- vs. bi-articular OA, but clinical observations support the idea that patients with OA in multiple large joints have a more severely compromised QoL and often require combined interventions for adequate improvement of functional status. The results of this study provide data in support of this perspective, highlighting the need for early identification of patients at risk of multi-articular involvement and the application of integrative therapeutic strategies. It is also important to differentiate polyarticular involvement from bilateral disease, which was prevalent in our cohort but not analyzed as a separate variable. While both conditions can increase mechanical stress and functional limitation, bilateral OA, such as in both knees or both hips, may lead to more symmetrical mobility deficits and a different pattern of compensation compared to combined hip and knee involvement. Although our methodology focused on the most severely affected joint, the high prevalence of bilateral OA likely contributes to the overall disability burden and should be considered in future analyses to better understand its independent impact on function and QoL.

In contrast to initial expectations and some reports in the literature, BMI was not a significant predictor of symptom severity in our groups. It is known that obesity increases the risk of OA onset and progression, with excess weight increasing mechanical stress on weight-bearing joints and favoring cartilage degradation [42]. Previous studies have also shown that obese patients with KOA tend to have worse WOMAC scores than normal-weight patients. Giotis et al. (2025) reported, for example, that an increased BMI was associated with significantly higher WOMAC scores (*p* = 0.023) in patients with knee pain [43]. In our group, however, the BMI–WOMAC correlation did not indicate significant results, and in the regression analysis the effect of BMI was insignificant. A plausible explanation for this discrepancy is the lack of variability; almost all our patients were overweight or obese (only <14% with BMI below 25), which made it difficult to highlight a gradient of severity according to weight. In practice, obesity became a background characteristic in our group rather than a discriminating factor.

Moreover, BMI does not differentiate between fat and lean body mass [44]. It is plausible that some male participants, particularly those with higher muscle mass, registered higher body weights that did not reflect excess adiposity, which would elevate BMI without a corresponding increase in symptom burden. Conversely, individuals with lower BMI may have presented with sarcopenia or frailty, potentially leading to worse functional scores despite having a lower body weight. The observed inverse trend might therefore reflect a complex interplay between muscle composition, metabolic status, and individual pain thresholds [45]. It also underscores the limitations of using BMI alone as a functional predictor, especially in clinical samples where body composition is not homogenous [46]. In this context, relying solely on BMI provides an incomplete picture.

Additionally, metabolic comorbidities such as diabetes mellitus, which were prevalent in the study cohort, may have influenced pain perception and functional reporting [47]. Reduced nociceptive sensitivity or neuropathic alterations in diabetic individuals could have resulted in underestimation of pain-related disability, further distorting the expected association potentially leading to an underestimation of pain and disability in self-reported instruments like WOMAC. Finally, it is important to note that the cross-sectional design of this study may have captured a temporary or atypical pattern that does not necessarily reflect the long-term trajectory of OA in relation to body weight. Without longitudinal follow-up, it is difficult to determine whether the observed associations represent stable trends or are influenced by short-term compensations, such as muscular adaptations or variations in health-seeking behavior. Recent data highlight that the impact of obesity and metabolic syndrome goes beyond simply stressing the joints, disrupting their normal function by altering tissue composition, promoting the polarization of inflammatory macrophages, and affecting chondrocyte metabolism. Synovial fluid in patients with KOA has been shown to promote the differentiation of monocytes into pro-inflammatory macrophages and to amplify the release of inflammatory mediators, thus perpetuating local inflammation and metabolic stress. These changes are accompanied by alterations in mitochondrial membrane potential and are suggestive of sustained cellular stress within the joint environment. Such findings support the view that metabolic dysregulation actively contributes to joint degradation by modulating immune responses and tissue behavior. Therefore, therapeutic approaches should not only address biomechanical load but also target the inflammatory and metabolic dimensions of the disease [48].

Regarding the staging of OA, we observed that patients in the end-stage (IV) did not present significantly higher WOMAC scores than those in progressive stage III, and in fact their average was slightly lower (50.8 vs. 53.4). This apparently counterintuitive result (theoretically, advanced radiological stage should correlate with more severe symptomatology) can be explained by several factors. First, the relatively small number of patients in advanced stages (*n* = 12) limits statistical power and causes the mean to be disproportionately influenced by individual variations. It is possible that some patients in the end-stage have adapted or have already undergone interventions (e.g., infiltrations, physiotherapy) that have partially improved their symptoms, maintaining the WOMAC score at a level comparable to those in moderate–severe stages. Secondly, it should be emphasized that the radiological stage does not always equate to the clinical stage, with some studies showing a discordance between the radiological image and the pain felt in patients with OA [49]. Some patients with advanced joint changes may have moderate pain, while others with less changes experience severe pain. Pain perception in OA is influenced by factors such as local inflammation, central pain sensitization, individual pain threshold, etc. [50]. An additional explanation may be found in the biological dynamics of late-stage OA itself. As cartilage becomes almost entirely eroded in the terminal phases of the disease, the release of inflammatory breakdown products, particularly glycosaminoglycans (GAGs), which are known to activate nociceptive signaling, diminishes substantially [51]. This reduction in molecular triggers may lead to a paradoxical decrease in inflammation and perceived pain, even though the joint damage is structurally severe. While the mechanical implications of cartilage loss remain profound, the biological drive for inflammation may actually decline in this final stage, offering a potential explanation for the mismatch between radiological severity and clinical symptoms observed in some patients Kulkarni et al. (2016) support this theory, suggesting that end-stage patients can be divided into two subgroups: one with high GAG levels, likely reflecting ongoing degradation, and another with severely depleted GAG levels (the so-called “floor effect”) where the cartilage is already absent, and the biochemical stimuli for inflammation are diminished [52].

Thus, the absence of a clear difference in WOMAC between PS and AS in our study does not invalidate the usefulness of staging but suggests that for the complete evaluation of the patient with OA, the radiological grade must be correlated with symptomatology and other clinical factors. In practice, a patient in the final radiological stage but with moderate symptoms is not uncommon, some of these patients choose to postpone prosthesis due to their clinical tolerance, while others in moderate stages but with intense pain may require early interventions [53]. This reinforces the importance of individualized care strategies that consider both structural damage and subjective experience.

In summary, our findings emphasize that joint-specific factors, namely the anatomical location and stage of OA, play a more central role in shaping functional outcomes and perceived QoL than broader demographic or systemic variables. The distinct impact of combined hip and knee involvement supports the need for individualized rehabilitation approaches that consider both joint distribution and disease progression. This study adds original evidence from a nationally underrepresented clinical context, reinforcing the importance of culturally and regionally tailored assessments in musculoskeletal rehabilitation. The use of validated tools in Romanian, WOMAC and WHOQOL-BREF, enabled a multidimensional evaluation, capturing not only functional disability but also the broader subjective burden experienced by patients.

Another contribution of the study is the highlighting of the fact that not all classic risk factors (such as obesity or female sex) uniformly influence clinical severity in a group of patients already diagnosed and treated for OA. This suggests that once the disease is established, management should be individualized. Not every obese patient with OA will necessarily have greater pain, although in the long term, weight loss remains essential for protecting joints. We also showed that patients with multi-joint impairment represent a vulnerable subgroup that could benefit from increased attention, namely aggressive pain management, a recovery plan tailored to multiple joints, as well as counseling for orthopedic interventions where necessary. These findings support the use of both objective and subjective outcome measures to capture the full extent of disease impact and guide therapeutic decision-making in OA.

Our research has several limitations that should be considered when interpreting the findings. First, the cross-sectional design restricts our ability to draw causal conclusions. While we identified associations between symptom severity and comorbidities, we cannot determine whether these conditions actively worsen OA or simply co-occur in patients with a higher overall disease burden. Second, all participants were recruited from a single rehabilitation center, which may limit the generalizability of the results to broader or more diverse populations. Patients presenting to rehabilitation services may differ in disease stage, health-seeking behavior, or comorbidity profile from those managed in primary care or surgical settings. Third, all data were collected via self-report questionnaires, which, although widely validated, are subject to recall bias and personal interpretation. We also did not assess the psychological dimensions such as depression or anxiety, factors known to influence both pain perception and QoL in patients with OA. Additionally, the study did not include performance-based measures of physical function or imaging evaluations beyond clinical staging, which could have added valuable objective context to the subjective data.

Future research should adopt longitudinal designs to track the evolution of symptoms and QoL over time, and to evaluate the impact of specific interventions. Investigating how tailored strategies, such as structured physical therapy, cognitive-behavioral approaches, or metabolic control, affect both functional outcomes and patient well-being would be especially valuable. Further, sex-specific analyses could shed light on differential coping styles or treatment responses, supporting the development of personalized, holistic approaches to managing hip and KOA.

## 5. Conclusions

The findings of this study support the hypothesis that HOA and KOA are associated with significant functional limitations and a moderate reduction in QoL. Although causality cannot be inferred from a cross-sectional design, the data suggest that patients with multi-joint involvement may require more comprehensive management strategies. Additionally, while no major sex differences were observed in symptom severity or QoL when adjusting for disease stage, advanced stages of OA and the presence of comorbidities were clearly linked to poorer outcomes. These insights emphasize the importance of individualized care approaches that integrate both physical and psychosocial dimensions of OA, tailored to the complexity of each patient’s clinical profile.

## Figures and Tables

**Table 1:** General characteristics of patients included in the study (*n* = 133).

Characteristic		Value (*n*, % or Mean ± SD)
Age (years), M ± SD		63.4 ± 9.8
Sex	Female	63 (47.4)
	Male	70 (52.6)
Residence	Urban area	95 (71.4)
	Rural area	38 (28.6)
Body mass index (BMI) M ± SD		32.2 ± 6.9 kg/m^2^
BMI ≥ 25 kg/m^2^ (overweight/obese)		115 (86.5)
IMC ≥ 30 kg/m^2^ (obese)		90 (67.7)
Comorbidities	Hypertension	106 (79.7)
	Type 2 DM	41 (30.8)
	CKD	26 (19.5)
	Peripheral varicose disease	65 (48.9)
	Other diagnoses	99 (74.43)
OA diagnosis	Isolated HOA	34 (25.6)
	Isolated KOA	32 (24.1)
	HOA + KOA	64 (50.4)
Severity stage	OA early stage (ES)	23 (17.3)
	OA progressive stage (PS)	98 (73.7)
	OA advanced/end-stage (AS)	12 (9.0)

BMI, body mass index; SD, standard deviation; IMC, corporal mass index; DM, diabetes mellites; CKD, chronic kidney disease; HOA, hip osteoarthritis; KOA, knee osteoarthritis; OA, osteoarthritis; ES, early stage; PS, progressive stage; AS, advanced stage.

**Table 2:** Mean values of WOMAC and WHOQOL-BREF scores at cohort level.

Parameter	Average ± SD	Median (Min–Max)
WOMAC total score (0–96)	52.0 ± 7.9	52 (36–67)
Pain (0–20)	7.8 ± 2.0	8 (4–15)
Stiffness (0–8)	5.8 ± 1.4	6 (2–8)
Physical function (0–68)	38.4 ± 5.6	38 (30–48)
WHOQOL-BREF total score	67.9 ± 13.1	72 (19–89)

WOMAC, Western Ontario and McMaster Universities Osteoarthritis Index; WHOQOL-BREF, World Health Organization Quality of Life-BREF.

**Table 3:** Distribution of OA and comorbidities by sex.

Category	Men, *n* (%)/ M ± SD	Women, *n* (%)/ M ± SD	*p* Value
Average age (years)	62.6 ± 10.64	64.3 ± 8.88	0.326
Average BMI (kg/m^2^)	32.54 ± 5.80	31.95 ± 7.77	0.327
Type 2 DM	26 (37.1)	28 (44.4)	0.392
Hypertension	54 (77.1)	53 (84.1)	0.311
CKD	16 (22.9)	10 (15.9)	0.311
Chronic venous insufficiency	36 (51.4)	29 (46.0)	0.534
Other associated diagnoses	51 (72.9)	48 (76.2)	0.098
HOA	48 (68.6)	50 (79.4)	0.158
KOA	54 (77.1)	42 (66.7)	0.983
HOA + KOA	34 (48.6)	30 (47.6)	0.392

*n*, number of patients; M, mean; SD, standard deviation; DM, diabetes mellitus; BMI, body mass index; CKD, chronic kidney disease; HOA, hip osteoarthritis; KOA, knee osteoarthritis.

**Table 4:** Mean values of the WOMAC total score and dimensions differentiated by sex.

WOMAC Score	Group	Mean ± SD	*p* Value
WOMAC/Pain	B	7.65 ± 1.89	0.795
	F	7.96 ± 2.03	
WOMAC/red stiffness	B	5.65 ± 1.54	0.131
	F	5.93 ± 1.33	
WOMAC/Function	B	37.81 ± 5.96	0.091
	F	39.00 ± 5.04	
WOMAC TOTAL	B	51.12 ± 8.41	0.102
	F	52.90 ± 7.26	
WHOQOL-BREF	B	69.3 ± 13.0	0.201
	F	66.4 ± 13.2	

WOMAC, Western Ontario and McMaster Universities Osteoarthritis Index; WHOQOL-BREF, World Health Organization Quality of Life-BREF.

**Table 5:** Multiple linear regression results for predicting WOMAC total score.

Predictor	β Coefficient	95% Confidence Interval	*p* Value
Age (years)	+0.039	−0.102 to +0.180	0.587
BMI (kg/m^2^)	−0.004	−0.193 to +0.186	0.970
Sex (female vs. male)	+0.592	−2.004 to +3.187	0.653
Combined Hip + Knee OA (No vs. Yes)	−4.987	−7.530 to −2.443	<0.001
Stage: Advanced/End-stage vs. Progressed	−2.275	−6.868 to +2.318	0.329
Stage: Early vs. Progressed	−6.346	−10.096 to −2.597	0.001
Type 2 DM (No vs. Yes)	+1.050	−2.051 to +4.150	0.504
Peripheral Varicose Disease (No vs. Yes)	+0.417	−2.269 to +3.104	0.759
Hypertension (No vs. Yes)	+1.486	−1.765 to +4.738	0.367
CKD (No vs. Yes)	+1.701	−1.805 to +5.207	0.339

β coefficients reflect the direction and magnitude of association with the total WOMAC score; CKD, Chronic Kidney Disease.

## Data Availability

The original contributions presented in the study are included in the article; further inquiries can be directed to the corresponding authors.

## Abbreviations

The following abbreviations are used in this manuscript:

OA	Osteoarthritis
KOA	Knee Osteoarthritis
HOA	Hip Osteoarthritis
QoL	Quality of Life
WHO	World Health Organization
WOMAC	Western Ontario and McMaster Universities Osteoarthritis Index
WHOQOL-BREF	World Health Organization Quality of Life-BREF version
BMI	Body Mass Index
